# The carbon and water footprints of data centers and what this could mean for artificial intelligence

**DOI:** 10.1016/j.patter.2025.101430

**Published:** 2025-12-17

**Authors:** Alex de Vries-Gao

**Affiliations:** 1 Institute for Environmental Studies, VU, Amsterdam, the Netherlands; 2 Digiconomist, Almere, the Netherlands; 3 De Nederlandsche Bank, Amsterdam, the Netherlands

**Keywords:** artificial intelligence, carbon footprint, water footprint

## Abstract

Although there are ways to estimate the global power demand of artificial intelligence (AI) systems, it remains challenging to quantify the associated carbon and water footprints. The lack of distinction between AI and non-AI workloads in the environmental reports of data center operators makes it possible to assess the environmental impact of AI workloads only by approximating them through data centers’ general performance metrics. The environmental disclosure of tech companies is, however, often insufficient to determine even the total data center performance of these companies. The shortcomings in the environmental disclosure of data center operators could be remedied with new policies mandating the disclosure of additional metrics. Because the environmental impact of data centers is growing rapidly, the urgency of transparency in the tech sector is also increasing. The carbon footprint of AI systems alone could be between 32.6 and 79.7 million tons of CO_2_ emissions in 2025, while the water footprint could reach 312.5–764.6 billion L.

## The bigger picture

Artificial intelligence (AI) systems are rapidly becoming the key growth driver of global data center electricity consumption. Despite AI system power demand approaching that of a country the size of the United Kingdom, the environmental impacts of this growth remain unclear. Most assessments focus on the cost of interacting with specific AI models but do not provide a more holistic overview. Such estimates are complicated by the fact that data center operators do not publicly disclose the required inputs. Reports that attempt to address the global environmental impact of AI hardware typically rely on proprietary analyst data, limiting validation in the public domain.

The lack of distinction between AI and non-AI workloads in the environmental reports of data center operators means it is possible to assess the environmental impact of AI workloads only by approximating them through data centers’ general performance metrics. Company-wide metrics from the environmental disclosure of data center operators suggest that AI systems may have a carbon footprint equivalent to that of New York City in 2025, while their water footprint could be in the range of the global annual consumption of bottled water. Further disclosures from data center operators are urgently required to improve the accuracy of these estimates and to responsibly manage the growing environmental impact of AI systems.

## Introduction

The release of OpenAI’s generative artificial intelligence (AI) ChatGPT at the end of 2022 triggered significant global growth in the demand for AI applications. Since then, the power demand of the hardware supporting these applications has been rising rapidly. The International Energy Agency (IEA) estimated that AI systems accounted for 15% of total data center electricity demand (excluding cryptocurrency mining) in 2024,^1^ and other recent research has suggested that AI systems represented 20% of total data center power demand by the end of 2024.^2^ Given the increasing manufacturing capacity of the AI system supply chain, the total power demand of these systems could reach 23 gigawatts by the end of 2025.^2^ This would mean that the share of AI system power demand would be almost half of the total data center electricity demand (47.4 gigawatts on average in 2024^1^), approaching as much as a country such as the United Kingdom requires (30.7 gigawatts on average in 2023^2^). This rising power demand could have significant environmental implications. For instance, the IEA estimated that in 2024, the electricity generation for global data centers could have produced approximately 182 million tons of CO_2_ emissions,^1^ and they were responsible for 560 billion L of water consumption in 2023.^1^

The carbon and water footprints of data centers are typically established by first assessing their locations and energy use, which are subsequently linked to characteristics of the respective electrical grids.^3^ The IEA, however, did not specify which parts of the estimated data center emissions and water consumption could be attributed to AI systems. The main challenge in determining such data is that even if a total AI power demand could be estimated, granular information regarding where AI systems are being operated cannot be obtained, despite past research emphasizing the importance of transparency in data centers^4^ and in information and communication technology (ICT) in general.^5^^,^^6^ This information is crucial to assess the carbon and water intensity of the electricity generation sources used to power this hardware. Tech companies such as Google, Microsoft, Meta, and Amazon have been identified as the largest buyers of AI systems,^4^ but their corporate sustainability reports do not specify the impacts associated with these systems. Without further input from data center operators, these facilities reveal nothing about the equipment they use.

This study explores the extent to which public sources can be used to provide direction for how the estimated power demand of AI systems translates to associated CO_2_ emissions and water consumption. Because operational emissions typically constitute the largest share of the life cycle emissions of data centers, the scope of this research is limited to operational impact. Embodied emissions represent 23% of the total footprint of ICT in general but less for data centers, specifically due to high electricity use.^6^ By highlighting the gaps in the available data and the potential scale of the environmental consequences of operating AI systems, the study could help policymakers understand both the urgency to promote transparency among data center operators and the key areas of concern.

## AI carbon footprint

Initially, the IEA’s findings on the average carbon intensity of electricity generation for global data centers appear to approximate the carbon intensity of generating electricity to run these centers’ AI systems. With an estimated 182 million tons of CO_2_ associated with 460 terawatt hours (TWh) of electricity generation for global data centers in 2024,^1^ the implied average carbon intensity per kilowatt hour (kWh) was 395.65 g of CO_2_ (gCO_2_/kWh). This figure is close to the IEA’s 2024 estimate of the global average emissions intensity from general electricity generation of 445 gCO_2_/kWh.^7^ The main reason for the lower carbon intensity of electricity generation for data centers is likely that they are primarily located in the United States (45%) and Europe (15%),^1^ whose regional carbon intensity of electricity generation on their power grids is approximately 321 and 174 gCO_2_/kWh, respectively.^7^

The IEA, however, mainly used proprietary datasets from research and intelligence companies such as IDC, Omdia, and SemiAnalysis on information technology (IT) equipment shipments to determine installed IT capacities and their regional distribution. Consequently, these figures cannot be easily validated. The dataset provided by the IEA contains only aggregated IT capacities per region, so this information cannot be used to connect specific data center locations to their respective power plants. This same process occurred in prior academic research by Siddik et al., revealing that the total electricity consumption of data centers in the United States was 72.15 TWh.^3^ This figure could be associated with 31.5 million tons of CO_2_ emissions, implying an average carbon intensity of 436.59 gCO_2_/kWh. Even so, the scope of this analysis was limited to the United States, and this work’s conclusion could be outdated because the only complete dataset Siddik et al. had access to was from 2018. A recent preprint by Guidi et al. attempted to update the work of Siddik et al., finding that United States data centers (excluding cryptocurrency miners) operating between September 2023 and August 2024 could have been responsible for 192.64 TWh of electricity consumption and 105.59 million metric tons of CO_2_,^8^ implying an average carbon intensity of 548.12 gCO_2_/kWh. Nevertheless, this upward trend was unexpected given the wider context of the global energy transition. Additionally, this trend was not explained by the authors in an article still requiring peer review, so this information might not be useful as a reference. Furthermore, even if a proper recent reference for data center carbon emissions in the United States could be found, the IEA only provides the regional breakdown of estimated IT capacities and does not replicate this for estimated carbon emissions, so a one-on-one comparison would still not be possible.

Currently, the United States dominates the IEA’s spatial distribution of data center capacity. Given the relatively low carbon intensity of electricity generation on this power grid, one could deduce that the carbon intensity of electricity generation for data centers in the United States must be lower than the IEA’s 2024 implied global average of 395.65 gCO_2_/kWh. An average carbon intensity of less than 395.65 gCO_2_/kWh would also be a logical development from 436.59 gCO_2_/kWh in 2018, considering the global energy transition, but more specific numbers have not been provided.

Beyond this, it is possible to examine the environmental reports of tech companies such as Google, Microsoft, Meta, and Amazon to ascertain how their data centers are performing. Although these companies do not provide AI-specific information, because they have been identified as the largest buyers of AI systems, their total data center environmental performance remains a useful approximation. Table 1 provides an overview of the relevant data that can be collected from the annual environmental reports of the tech companies identified as significant buyers of AI systems (Data S1, sheet 1).

**Table 1:** Availability of environmental performance metrics in sustainability reports of selected companies

Reporting metric	Reporting scope	Company (year of most recent environmental report publication) and reporting period										
		Amazon (2025)	Apple (2025)	Baidu (2025)	ByteDance (N/A^a^)	CoreWeave (N/A^a^)	Google (2025)	Meta (2025)	Microsoft (2025)	Oracle (2025)	Tesla (2025)	Tencent (2025)
		CY 2024	FY 2024	CY 2024			CY 2024	CY 2024	FY 2024	CY 2023	CY 2024	CY 2024
Electricity consumption	total company	no	yes	yes	no	no	yes	yes	yes	yes	yes	yes
	total data centers	no	yes	no	no	no	yes	yes	no	no	no	no
	individual data centers	no	yes	no	no	no	no	yes	no	no	no	no
	AI workloads	no	no	no	no	no	no	no	no	no	no	no
Scope-2 emissions (location based)	total company	yes	yes	yes	no	no	yes	yes	yes	yes	yes	yes
	total data centers	no	no	no	no	no	no	yes	no	no	no	no
	individual data centers	no	no	no	no	no	no	yes	no	no	no	no
	AI workloads	no	no	no	no	no	no	no	no	no	no	no
Scope-2 emissions (market based)	total company	yes	yes	yes	no	no	yes	yes	yes	yes	yes	yes
	total data centers	no	yes	no	no	no	no	yes	no	no	no	no
	individual data centers	no	yes	no	no	no	no	yes	no	no	no	no
	AI workloads	no	no	no	no	no	no	no	no	no	no	no
Direct water consumption	total company	no	yes	yes	no	no	yes	yes	yes	yes	no	yes
	total data centers	WUE only	no	WUE only	no	no	yes	yes	WUE only	no	no	no
	individual data centers	no	no	no	no	no	yes	no	no	no	no	no
	AI workloads	no	no	no	no	no	no	no	no	no	no	no
Indirect water consumption	total company	no	no	no	no	no	no	yes	no	no	no	no
	total data centers	no	no	no	no	no	no	no	no	no	no	no
	individual data centers	no	no	no	no	no	no	no	no	no	no	no
	AI workloads	no	no	no	no	no	no	no	no	no	no	no

Assessment is based on the most recent available environmental report on October 19, 2025. Companies may report data over calendar years (CY, Jan. 1–Dec. 31) or fiscal years (FY). In the latter case, the reported data does not correspond to a calendar year, as companies may choose the start and end dates. Apple’s fiscal year 2024 ended on September 28, 2024. Microsoft’s fiscal year 2024 ended on June 30, 2024.

a Company does not publish environmental reports.

Table 1 is partially based on Masanet et al.,^4^ who introduced a bottom-up model for estimating the electricity consumption and onsite water usage of AI data centers. In their framework, Masanet et al. examined whether data center operators disclosed electricity use, power usage effectiveness (PUE; a measure for data center power efficiency), and water usage effectiveness (WUE; a measure for data center water efficiency). While environmental impacts such as carbon emissions and indirect water consumption could be modeled given sufficiently granular disclosure of the aforementioned metrics, the absence of such granular data in most current environmental reports (Table 1) forces the inclusion of other metrics that can be helpful in assessing the current environmental performance of AI data centers. Table 1 therefore addresses the availability of reporting metrics for (location-based and market-based) scope-2 emissions (which capture the carbon emissions of purchased electricity) and (in)direct water consumption, as these metrics directly reflect the environmental impacts in this study’s scope.

In the table, environmental disclosure is both limited and inconsistent across the companies examined. No company reports any AI-specific metrics, although firms such as Google, Meta, and Microsoft report significant increases in electricity consumption in 2023 and 2024, attributing this growth to AI. Google also mentions AI as a key driver of its power demand growth: “As AI and other technologies expand to unlock new economic and social benefits, the demand for digital services has grown rapidly, which in turn creates demand for data centers that require increased energy for operations and water for cooling.”^9^ Microsoft states, “AI workloads drive increased compute resource needs,”^10^ while Meta reports, “The challenge of reaching our sustainability goals given the increased demand for energy and resources driven by AI is not unique to Meta.”^11^

Of the companies in Table 1**,** Meta provides the most extensive disclosure. It not only distinguishes between its total electricity consumption (and associated carbon emissions) and the electricity consumption of only its data centers but also reports these metrics for individual data centers.^11^ Apple provides a similar breakdown of data center locations and electricity consumption but provides only market-based and not location-based carbon emissions.^12^ In contrast, Google provides only water consumption metrics per individual data center.^9^

In other cases, most of the companies examined specify only their total performance, so it is unclear which part could be attributed to data centers. Notably, Amazon, despite reporting the largest location-based scope-2 emissions of all the companies,^13^ does not disclose its electricity consumption,^14^ and ByteDance and CoreWeave do not provide any environmental disclosure. Table 2 summarizes the electricity consumption and carbon emission data available from the environmental reports.

**Table 2:** Reported electricity consumption and location-based scope-2 emissions in sustainability reports of selected companies

Reporting metric	Reporting scope	Unit	Company (year of most recent environmental report publication) and reporting period								
			Amazon (2025)	Apple (2025)	Baidu (2025)	Google (2025)	Meta (2025)	Microsoft (2025)	Oracle (2025)	Tesla (2025)	Tencent (2025)
			CY 2024	FY 2024	CY 2024	CY 2024	CY 2024	FY 2024	CY 2023	CY 2024	CY 2024
Electricity consumption	total company	MWh	–	3,776,000	1,207,350	32,106,200	18,423,634	29,829,540	3,623,170	2,355,046	6,429,610
	total data centers	MWh	–	2,515,000	–	30,825,600	18,061,781	–	–	–	–
Scope-2 emissions (location based)	total company	tCO_2_	17,760,000	1,224,500	777,439	11,283,200	5,967,348	9,955,368	1,149,400	754,000	3,642,095
	total data centers	tCO_2_	–	–	–	–	5,862,615	–	–	–	–
Implied carbon intensity^a^	total company	tCO_2_/MWh	–	0.32	0.64	0.35	0.32	0.33	0.32	0.32	0.57
	data centers only	tCO_2_/MWh	–	–	–	–	0.32	–	–	–	–

Companies may report data over calendar years (CYs; January 1–December 31) or fiscal years (FYs). In the latter case, the reported data do not correspond to a CY, as companies may choose the start and end dates. Apple’s FY 2024 ended on September 28, 2024. Microsoft’s FY 2024 ended on June 30, 2024. Apple, Google, and Meta are the only companies reporting electricity consumption for both the total company and data centers. In these cases, data centers respectively represent 67%, 96%, and 98% of total electricity consumption. Likewise, data center carbon emissions at Meta represent 98% of total company carbon emissions. Implied carbon intensity is obtained by dividing the reported carbon emissions by the reported electricity consumption. −, value is not reported/cannot be determined.

a Amount of reported scope-2 carbon emissions per unit of reported electricity consumption.

For the companies included in Table 2 that provide both electricity consumption and location-based scope-2 emissions, the combined total electricity consumption equals 97.8 TWh. The same companies report a total of 34.8 million tons of associated carbon emissions, implying a weighted average carbon intensity of 355.53 gCO_2_/kWh (Data S1, sheet 2). This figure can only be established at a total company level since the combination of both electricity consumption and location-based scope 2 emissions data is rarely specified for these companies’ data centers. Meta is the only one that provides both of these metrics, highlighting that data centers represent most (98%) of the company’s electricity consumption and carbon emissions. The carbon intensity of electricity consumed by Meta’s data centers therefore matches that of its total electricity consumption.

Google’s situation is likely similar, as data centers represent a majority (96%) of the company’s electricity consumption, but an exact figure cannot be determined due to the absence of reporting of carbon emissions related to data centers. Moreover, Apple illustrates why performance metrics at a total company level may not be representative of the environmental performance of a company’s data centers. In Apple’s case, data center electricity consumption represents two-thirds of the company’s total electricity consumption, as the company, for example, also operates a network of retail stores. The environmental impact of this global network of retail stores could deviate from the impact of the company’s data centers, which are concentrated in the United States.

At the same time, it must be established that performance metrics at a total company level are currently the best available reference for the environmental performance of data center operators. The American companies Apple, Google, Meta, Microsoft, Oracle,^15^ and Tesla^16^ show similar carbon intensities of electricity consumed, ranging from 0.32 to 0.35 tCO_2_/MWh. This range is slightly lower than the carbon intensity (395.65 gCO_2_/kWh) of electricity generation for data centers in 2024 found by the IEA.^1^ These companies primarily operate data centers in the United States and Europe, regions with relatively green power grids, and should be considered the best in class in data center performance metrics.

The data from Chinese companies Baidu^17^ and Tencent,^18^ with carbon intensities of electricity consumed equal to 0.64 and 0.57 tCO_2_/MWh, respectively, suggest that the same metrics for data centers outside the United States and Europe are likely to be higher. Overall, the IEA’s estimated carbon intensity (395.65 gCO_2_/kWh) of electricity generation for data centers in 2024 appears plausible.

One can apply this carbon intensity to the estimated power demand of AI systems of 9.4 GW at the end of 2024 and a potential increase to 23 GW through 2025,^2^ revealing that AI systems could be responsible for 32.6–79.7 million tons of CO_2_ emissions in 2025 (Data S1, sheet 4). Nevertheless, significant uncertainty surrounds these figures. In the United States alone, the power grids supporting the specified data center locations of Apple, Google, and Meta can be associated with carbon intensities ranging from 0.17 to 0.46 tCO_2_/MWh (Data S1, sheet 3). Therefore, while data center averages may be useful starting points for assessing the environmental impact of AI systems, there remains a clear need for more granular disclosure.

## AI water footprint

The water footprint of AI is even more difficult to assess than its carbon footprint because a company’s total water consumption (i.e., water that becomes unavailable for reuse after withdrawing it from a water supply) comprises both direct and indirect use. Direct water consumption is the water consumed in the on-site cooling of data centers, whereas indirect water consumption occurs in the process of generating electricity for data centers. The IEA estimated that the total water consumption of data centers in 2023 totaled 560 billion L, with two-thirds (373 billion L) relating to indirect water consumption and only a quarter (140 billion L) to direct water consumption.^1^ The remainder of the estimate (about 8%) involves water consumption in hardware manufacturing (47 billion L), which is outside of this study’s scope (though in line with the observation that operational impacts typically represent a majority of a data center’s total life cycle impact^6^).

The water intensity of electricity generation could, however, range from less than a liter to thousands of liters per kWh generated, depending on the generation technology and location.^19^ Additionally, tech companies, at best, only disclose their direct water consumption (see Table 1), making it challenging to verify the IEA’s output. Table 3 summarizes the electricity and water consumption data from the environmental reports of the tech companies listed in Table 1.

**Table 3:** Reported electricity consumption and (in)direct water consumption in sustainability reports of selected companies

Reporting metric	Reporting scope	Unit	Company (year of most recent environmental report publication) and reporting period								
			Amazon (2025)	Apple (2025)	Baidu (2025)	Google (2025)	Meta (2025)	Microsoft (2025)	Oracle (2025)	Tesla (2025)	Tencent (2025)
			CY 2024	FY 2024	CY 2024	CY 2024	CY 2024	FY 2024	CY 2023	CY 2024	CY 2024
Electricity consumption	total company	MWh	–	3,776,000	1,145,028	32,106,200	18,423,634	29,829,540	3,623,170	2,355,046	6,429,610
	total data centers	MWh	–	2,515,000	–	30,825,600	18,061,781	–	–	–	–
	data center average PUE	N/A	1.15	–	1.20	1.09	1.08	1.16	–	–	1.29
Direct water consumption	total company	m^3^	–	3,406,871	1,465,129	30,794,325	3,123,000	5,807,000	866,069	–	10,370,588
	total data centers	m^3^	–	–	–	29,477,002	2,974,000	–	–	–	–
	data center average WUE	m^3^/MWh	0.15	–	1.61	–	0.19	0.30	–	–	–
Indirect water consumption	total company	m^3^	–	–	–	–	72,207,000	–	–	–	–
	total data centers	m^3^	–	–	–	–	–	–	–	–	–
Implied water intensity^a^	total company (direct)	m^3^/MWh	–	0.90	1.28	0.96	0.17	0.19	0.24	–	1.61
	total company (indirect)	m^3^/MWh	–	–	–	–	3.92	–	–	–	–

Companies may report data over calendar years (CYs; January 1–December 31) or fiscal years (FYs). In the latter case, the reported data do not correspond to a CY, as companies may choose the start and end dates. Apple’s FY 2024 ended on September 28, 2024. Microsoft’s FY 2024 ended on June 30, 2024. Apple, Google, and Meta are the only companies reporting electricity consumption for both the total company and data centers. In these cases, data centers respectively represent 67%, 96%, and 98% of total electricity consumption. Likewise, direct data center water consumption at Google and Meta, respectively, represent 96% and 95% of total company direct water consumption. Implied water intensity is obtained by dividing the reported water consumption by the reported electricity consumption. −, value is not reported/cannot be determined.

a Amount of reported water consumption per unit of reported electricity consumption.

Table 3 illustrates that Meta is the only company reporting “water consumption embedded in purchased electricity”^11^ (i.e., indirect water consumption) while also highlighting that this value is significantly higher than Meta’s direct water consumption. For the companies included in Table 3 that provide both electricity and direct water consumption data, the total direct water consumption is 55.8 million m^3^ (55.8 billion L). For a total electricity consumption of 97.8 TWh, there is a weighted average of 0.59 L per kWh consumed. The latter figure may be the best available reference for the water efficiency of these companies’ data centers. This efficiency in the context of data centers is normally captured by the metric WUE, expressing the total on-site water consumption relative to the electricity consumption of the IT equipment. The equation for calculating WUE is as follows:

(Equation 1) $$\text{WUE}=\frac{\text{direct}\;\text{water}\;\text{consumption}\;\left({\text{million}\;m}^{3}\right)}{\text{IT}\;\text{equipment}\;\text{electricity}\;\text{consumption}\;\left(\text{TWh}\right)}\text{.}$$

If only the total data center electricity consumption is known, the electricity consumption of the IT equipment can be determined, as long as the average PUE is provided. PUE expresses the total facility electricity consumption relative to the electricity consumption of the IT equipment. This can be written as follows:

(Equation 2) $$\text{PUE}=\frac{\text{total}\;\text{facility}\;\text{electricity}\;\text{consumption}\;\left(\text{TWh}\right)}{\text{IT}\;\text{equipment}\;\text{electricity}\;\text{consumption}\;\left(\text{TWh}\right)}\text{.}$$

To determine IT equipment electricity consumption when only the total data centers’ electricity consumption and PUE values are provided, the previous equation (Equation 2) should be rearranged as follows:

(Equation 3) $$\text{IT}\;\text{equipment}\;\text{electricity}\;\text{consumption}\;\left(\text{TWh}\right)=\frac{\text{total}\;\text{facility}\;\text{electricity}\;\text{consumption}\;\left(\text{TWh}\right)}{\text{PUE}}\text{.}$$

Even so, although several tech companies disclose the WUE and PUE values for their data centers, too few of these enterprises report their data center electricity consumption, meaning a broad weighted-average water intensity of electricity consumption can be determined only at a total company level. The IEA reported about 140 billion L of direct water consumption on a total data center electricity consumption of 360 TWh and a global average PUE of 1.43 in 2023,^1^ making the implied WUE 0.56. This follows from first establishing IT equipment electricity consumption using Equation 3:

$$\frac{\text{total}\;\text{facility}\;\text{electricity}\;\text{consumption}\;\left(\text{TWh}\right)}{\text{PUE}}=\frac{360}{1.43}=251.75\;\text{TWh,}$$

which, using Equation 1, gives a WUE as follows:

$$\frac{\text{direct}\;\text{water}\;\text{consumption}\;\left({\text{million}\;m}^{3}\right)}{\text{IT}\;\text{equipment}\;\text{electricity}\;\text{consumption}\;\left(\text{TWh}\right)}=\frac{140}{251.75}=0.56\text{.}$$

This figure is near the weighted average of 0.59 L per kWh consumed reported by tech companies in 2024. The IEA’s estimated direct water consumption of data centers therefore seems plausible.

That is not the case for the IEA’s indirect water consumption assessment. With an estimated 373 billion L of indirect water consumption for an estimated electricity consumption of 360 TWh, the average water intensity of electricity generated to power data centers would be 1.04 L per kWh. This figure is just double the direct water consumption factor of data centers, but a more significant difference could be expected.

For a start, Siddik et al. reported that in 2018, the total indirect water consumption of data centers in the United States was 383 billion L. For an estimated electricity consumption of 72.15 TWh over the same time period,^3^ the average water intensity was 5.3 L per kWh consumed. The average water intensity may have decreased since then but probably not by as much as implied by the IEA, based on Meta’s water consumption disclosure, shown in Table 3. Meta reported a total indirect water consumption of 72.2 billion L for 18.4 TWh of electricity consumption in 2024,^20^ averaging 3.92 L per kWh. This figure was almost four times the IEA’s average water intensity of data center electricity consumption.

As the IEA estimated the carbon footprint of data centers in 2024 and their water footprint in 2023,^1^ one can obtain metrics from prior years for a more direct comparison of the water footprint. Meta reported a total indirect water consumption of 55.5 billion L for 15.3 TWh of electricity consumption in 2023, averaging to 3.62 L per kWh.^11^ This figure was still about 3.5 times the IEA’s average water intensity of data center electricity consumption. Meta detailed their electricity consumption per data center, so it is possible to match the electricity consumption in these locations with the water and carbon intensity factors of the respective power grids obtained from the Lawrence Berkeley National Laboratory.^21^ Doing so reveals that Meta’s United States data centers could account for 37.2 billion L of water consumption for 10.6 TWh of electricity consumption in 2023, averaging 3.50 L per kWh given the water intensity of electricity consumption in each region (Data S1, sheet 3).

Notably, the estimated indirect water consumption of these data centers (37.2 billion L) represented 67% of Meta’s reported total indirect water consumption, in line with the total electricity consumption of the selected data centers, as these represented 69% of Meta’s reported total electricity consumption. Apple does not report its indirect water consumption, but like Meta, the company does report electricity consumption per data center. Therefore, this information was used to determine that Apple’s United States data centers were likely responsible for 13.0 billion L of water consumption for an electricity consumption of 1.7 TWh in 2023, averaging out to 7.86 L per kWh (Data S1, sheet 3). Table 4 summarizes the environmental performance of Apple’s and Meta’s individual United States data center locations in 2023.

**Table 4:** Reported electricity consumption and estimated associated scope-2 emissions and water consumption for individual United States data center locations of Apple and Meta (2023)

Company	Location	Reported electricity consumption (MWh)	Grid tCO_2_/MWh	Grid L/kWh	Scope-2 CO_2_ (tCO_2_)	Embedded water (m^3^)
Apple	Maiden, NC	453,000	0.27	11.98	122,310	5,426,940
	Mesa, AZ	488,000	0.21	2.84	102,480	1,385,920
	Prineville, OR	269,000	0.36	10.69	96,840	2,875,610
	Reno, NV	440,000	0.34	7.46	149,600	3,282,400
	total	1,650,000	0.29	7.86	471,230	12,970,870
Meta	Altoona (IA)	1,243,306	0.35	1.74	435,157	2,163,352
	DeKalb (IL)	138,965	0.35	1.74	48,638	241,799
	Eagle Mountain (UT)	787,740	0.2	1.25	157,548	984,675
	Forest City (NC)	507,068	0.46	3.21	233,251	1,627,688
	Fort Worth (TX)	1,029,570	0.31	0.68	319,167	700,108
	Gallatin (TN)	116,520	0.46	3.21	53,599	374,029
	Henrico (VA)	805,061	0.35	1.74	281,771	1,400,806
	Huntsville (AL)	614,198	0.25	2.8	153,550	1,719,754
	Los Lunas (NM)	1,110,100	0.27	0.74	299,727	821,474
	New Albany (OH)	793,063	0.46	3.21	364,809	2,545,732
	Prineville (OR)	1,375,321	0.37	11.3	508,869	15,541,127
	Sarpy (NE)	1,148,091	0.39	2.77	447,755	3,180,212
	Stanton Springs (GA)	968,565	0.38	6.08	368,055	5,888,875
	total	10,637,568	0.35	3.50	3,671,896	37,189,633

Local grid carbon and water intensity factors were obtained from the Lawrence Berkeley National Laboratory.

Lastly, although Google discloses neither its indirect water consumption nor the electricity consumption per data center, it does disclose its direct water consumption per data center. Because one could obtain a WUE factor for Google’s data centers using the company’s reported data center electricity consumption, PUE, and direct water consumption (Table 3), this value may be used to determine the electricity consumption of individual data centers and thus the associated indirect water consumption. Using Equation 3, Google’s IT equipment electricity consumption is determined as follows:

$$\frac{\text{total}\;\text{facility}\;\text{electricity}\;\text{consumption}\;\left(\text{TWh}\right)}{\text{PUE}}=\frac{30.83}{1.09}=28.28\;\text{TWh.}$$

Google’s data center average WUE (using Equation 1) must therefore be

$$\frac{\text{direct}\;\text{water}\;\text{consumption}\;\left({\text{million}\;m}^{3}\right)}{\text{IT}\;\text{equipment}\;\text{electricity}\;\text{consumption}\;\left(\text{TWh}\right)}=\frac{29.48}{28.28}=1.04\text{.}$$

Assuming each of Google’s data centers consumes 1.04 L of water for every kWh consumed, the total direct water consumption of its United States data centers (18.3 billion L) translates to an estimated electricity consumption of 19.4 TWh in 2023, as shown in Table 5. Notably, this also assumes a fixed PUE of 1.10, equivalent to the company’s reported data center average in 2023,^22^ for each location. Although Google specifies PUE values for individual data center locations as well, this specification does not perfectly align with its water consumption disclosure for individual data center locations; therefore, the company’s data center average PUE is used instead.

**Table 5:** Reported water consumption and estimated electricity consumption, along with associated scope-2 emissions and water consumption, for individual Google United States data center locations (2023)

Location	Reported water consumption (m^3^)	Assumed WUE	Assumed PUE	Estimated electricity consumption (MHh)	Grid tCO_2_/MWh	Grid L/kWh	Scope-2 CO_2_ (tCO_2_)	Embedded water (m^3^)
Ashburn, VA	206,683	1.04	1.10	218,122	0.35	1.74	76,343	379,533
Berkeley County, SC	2,889,783	1.04	1.10	3,049,718	0.35	1.74	1,067,401	5,306,510
Council Bluffs, IA	3,710,082	1.04	1.10	3,915,416	0.46	3.21	1,801,091	12,568,486
The Dalles, OR	1,144,709	1.04	1.10	1,208,062	0.37	11.3	446,983	13,651,104
Douglas County, GA	1,308,238	1.04	1.10	1,380,643	0.46	3.21	635,096	4,431,863
Henderson, NV	601,123	1.04	1.10	634,393	0.31	0.68	196,662	431,387
Jackson County, AL	538,286	1.04	1.10	568,077	0.31	0.68	176,104	386,292
Lancaster, OH	29,148	1.04	1.10	30,761	0.35	1.74	10,766	53,524
Leesburg, VA	655,633	1.04	1.10	691,919	0.35	1.74	242,172	1,203,940
Lenoir, NC	1,274,927	1.04	1.10	1,345,487	0.17	3.67	228,733	4,937,939
Lockbourne, OH	88,200	1.04	1.10	93,082	0.35	1.74	32,579	161,962
Mayes County, OK	3,085,489	1.04	1.10	3,256,255	0.39	2.77	1,269,940	9,019,827
Midlothian, TX	514,059	1.04	1.10	542,509	0.35	1.74	189,878	943,966
Montgomery County, TN	1,092,470	1.04	1.10	1,152,932	0.31	0.68	357,409	783,994
New Albany, OH	481,126	1.04	1.10	507,754	0.46	3.21	233,567	1,629,889
Papillion, NE	509,895	1.04	1.10	538,115	0.39	2.77	209,865	1,490,579
Sterling, VA	210,469	1.04	1.10	222,117	0.31	0.68	68,856	151,040
Storey County, NV	757	1.04	1.10	799	0.35	7.63	280	6,096
Total	18,341,077	1.04	1.10	19,356,162	0.37	2.97	7,243,724	57,537,931

Local grid carbon and water intensity factors were obtained from the Lawrence Berkeley National Laboratory.

The water and carbon intensities of the power grids of Google’s United States-based data centers could indicate that the associated carbon emissions amounted to 7.2 million tons of CO_2_, while the embedded water consumption could be approximately 57.5 billion L. Thus, an average of 2.97 L per kWh would be consumed. Notably, this estimation is uncertain because the true WUE per individual data center is not provided by Google, but the estimated carbon footprint of these data centers closely aligns with Google’s reported scope-2 carbon emissions of 6.9 million tons of CO_2_ for the North America region.

Overall, the combined United States-based data centers of Meta, Apple, and Google may have caused 31.7 TWh of electricity consumption, which could be associated with 107.7 billion L of indirect water consumption in 2023. Consequently, the weighted-average water intensity of these companies’ United States-based data centers would be 3.40 L per kWh (or 4.08 L when considering only Meta and Apple data). These data would be closer to the findings of Siddik et al. than the water intensity estimated by the IEA in 2023.

One could apply an estimated water intensity of 3.40 L per kWh to the IEA’s estimated data center electricity consumption of approximately 360 TWh in 2023, producing an estimated 1,225 billion L of indirect water consumption on top of the estimated 140 billion L of direct water consumption. Thus, based on available sustainability reporting, the IEA could be significantly underestimating the water footprints of data centers.

Notably, the Berkeley Lab also published a report on the energy usage of data centers in the United States, using similar proprietary sources to the IEA. It found that United States data centers were responsible for an indirect water consumption of 800 billion L for 176 TWh of electricity consumption in 2023, averaging out to 4.52 L per kWh.^23^ This further supports the notion that the IEA could be underestimating the water footprint of data centers.

Considering the estimated power demand of AI systems of 9.4 GW at the end of 2024 and the potential increase to 23 GW through 2025, the total water footprint of AI systems alone could reach between 312.5 and 764.6 billion L in 2025 (Data S1, sheet 4). These results would mean that water intensity factors remained constant. At the same time, the uncertainty surrounding these figures would also remain significant because the power grids supporting the specified United States data center locations of Apple, Google, and Meta can be associated with water intensities ranging from 0.68 to 11.98 L/kWh (Data S1, sheet 3). Ultimately, further disclosures from data center operators are required to improve the accuracy of these estimates.

## Discussion and conclusion

Although there are ways to estimate the global power demand of AI systems, it remains challenging to quantify the associated carbon and water footprints. The lack of distinction between AI and non-AI workloads in the environmental reports of data center operators means it is only possible to assess the environmental impact of AI workloads by approximating them through data centers’ general performance metrics. The environmental disclosure of tech companies is, however, often insufficient to assess even the total data center performance of these companies.

In such cases, company-wide metrics may be the best available information, but these values may not accurately represent the data center performance, depending on the (undisclosed) weight of the data centers within the respective companies’ operations. Furthermore, company-wide metrics are not always available, and some companies do not disclose any environmental impact data. Specifically, embedded water consumption in purchased electricity is rarely disclosed at any level.

Recently, Google published a report on the environmental impact of its Gemini AI model,^24^ commenting that it chose not to report indirect water consumption because it “does not fully control the water consumption in electricity generation.”^25^ This statement highlights a lack of willingness to disclose this metric, ignoring that this water consumption is a direct consequence of Google’s electricity demand. For the same reason, the Greenhouse Gas Protocol already mandates disclosure of indirect emissions related to electricity consumption.^26^

The shortcomings in the environmental disclosure of data center operators could be remedied with new policies mandating the disclosure of additional metrics. Because the environmental impact of data centers is growing rapidly, the urgency for transparency in the tech sector is also increasing. The carbon footprint of AI systems alone could be between 32.6 and 79.7 million tons of CO_2_ emissions in 2025, while the water footprint could reach 312.5–764.6 billion L. To put this into perspective, this is in the same range as the carbon footprint of New York City (52.2 million tons of CO_2_ emissions in 2023^27^). Similarly, the water footprint of AI systems may be in the same range as the entire global annual consumption of bottled water (446 billion L^28^). This assessment can be made only by using the wider data center performance metrics in the environmental reports of data center operators, so significant uncertainty surrounds these figures.

There is strong variation in the carbon and water intensity of local power grids; thus, estimation accuracy would greatly improve if companies disclosed the specific locations where AI systems are operated and the magnitudes of these operations. Additionally, PUE and WUE values should be disclosed for each of these locations.

Microsoft’s 2025 environmental report noted that the company “launched a new datacenter design that optimizes AI workloads and uses zero water for cooling.”^10^ Consequently, the direct water consumption profile of data centers operating AI systems may also differ from those facilitating other types of workloads, but these design differences remain unclear without further transparency. Moreover, the lack of transparency makes it difficult to assess the potential effectiveness of mitigation strategies outlined by past research. Computation may be made more sustainable by rethinking how digital systems are designed, built, and operated,^5^ supported by policy mechanisms that enforce sector-wide climate targets.^6^ However, without transparent data, the biggest opportunities for mitigating the climate impacts of data centers and AI cannot be easily identified, and the effects of interventions will remain hidden as well. If transparency improved, future research could explore beyond the environmental impact of operating AI systems by examining the full life cycle impact of the relevant hardware.

## Declaration of interests

The author declares no competing interests.
